# Development and validation of multivariable prediction models of serological response to SARS-CoV-2 vaccination in kidney transplant recipients

**DOI:** 10.3389/fimmu.2022.997343

**Published:** 2022-10-04

**Authors:** Bilgin Osmanodja, Johannes Stegbauer, Marta Kantauskaite, Lars Christian Rump, Andreas Heinzel, Roman Reindl-Schwaighofer, Rainer Oberbauer, Ilies Benotmane, Sophie Caillard, Christophe Masset, Clarisse Kerleau, Gilles Blancho, Klemens Budde, Fritz Grunow, Michael Mikhailov, Eva Schrezenmeier, Simon Ronicke

**Affiliations:** ^1^ Department of Nephrology and Medical Intensive Care, Charité–Universitätsmedizin Berlin, corporate member of Freie Universität Berlin, Humboldt-Universität zu Berlin, and Berlin Institute of Health, Berlin, Germany; ^2^ Department of Nephrology, Medical Faculty, University Hospital Düsseldorf, Heinrich-Heine-University, Düsseldorf, Germany; ^3^ Division of Nephrology and Dialysis, Department of Internal Medicine III, Medical University Vienna, Vienna, Austria; ^4^ Department of Nephrology and Transplantation, University Hospitals of Strasbourg, INSERM Unit 1109, Strasbourg, France; ^5^ Institut de Transplantation Urologie Néphrologie, Centre Hospitalier Universitaire de Nantes, Centre de Recherche en Transplantation et Immunologie, UMR 1064, INSERM, Nantes Université, Nantes, France; ^6^ Berlin Institute of Health, Berlin, Germany

**Keywords:** kidney transplantation, COVID-19, vaccination, clinical decision support, immunosuppression therapy

## Abstract

Repeated vaccination against SARS-CoV-2 increases serological response in kidney transplant recipients (KTR) with high interindividual variability. No decision support tool exists to predict SARS-CoV-2 vaccination response to third or fourth vaccination in KTR. We developed, internally and externally validated five different multivariable prediction models of serological response after the third and fourth vaccine dose against SARS-CoV-2 in previously seronegative, COVID-19-naïve KTR. Using 20 candidate predictor variables, we applied statistical and machine learning approaches including logistic regression (LR), least absolute shrinkage and selection operator (LASSO)-regularized LR, random forest, and gradient boosted regression trees. For development and internal validation, data from 590 vaccinations were used. External validation was performed in four independent, international validation cohorts comprising 191, 184, 254, and 323 vaccinations, respectively. LASSO-regularized LR performed on the whole development dataset yielded a 20- and 10-variable model, respectively. External validation showed AUC-ROC of 0.840, 0.741, 0.816, and 0.783 for the sparser 10-variable model, yielding an overall performance 0.812. A 10-variable LASSO-regularized LR model predicts vaccination response in KTR with good overall accuracy. Implemented as an online tool, it can guide decisions whether to modulate immunosuppressive therapy before additional active vaccination, or to perform passive immunization to improve protection against COVID-19 in previously seronegative, COVID-19-naïve KTR.

## Introduction

SARS-CoV-2 vaccination offers protection from severe coronavirus disease 2019 (COVID-19) regardless of the causative variant for most healthy individuals. (1) In contrast, protection in immunocompromised solid organ transplant (SOT) recipients is limited. The serological response rate after SARS CoV-2 vaccination in kidney transplant recipients (KTR) after three doses of vaccine is strongly impaired in comparison to the general population – resulting in insufficient protection and an unacceptably high COVID-19 mortality within this population (2, 3).

Different strategies to induce humoral protection for KTR have been suggested, including repeated vaccination and vaccination under adjusted immunosuppression – besides SARS-CoV-2-specific monoclonal antibody therapy (4). Existing data are helpful to identify factors associated with insufficient vaccination response, but are not easily interpretable for the single patient or vaccination (5–7). Specifically, no tool exists to predict individual response to a vaccination. Risk calculators can help assess the likelihood of vaccination success in an individual and help decide between different possible actions such as passive or active immunization or adjustment of immunosuppressive medication. To date, no such decision support system is available.

For this reason, we aim to develop a classification model to predict serological response to third and fourth SARS-CoV-2 vaccinations in previously seronegative, COVID-19-naïve KTR. The model’s implementation objective is to identify patients that will likely not respond to an additional dose of vaccine, even with changes in immunosuppressive medication, and thus benefit most from passive immunization strategies. Using our previously reported data of vaccination outcomes in KTR, we develop and compare a set of prediction models based on classical statistical methods as well as machine learning. After selecting the most promising models, we validate the resulting prediction models in four independent validation cohorts, and make the result available as an online calculator.

## Methods

### Development cohort

Data from KTR at Charité – Universitätsmedizin Berlin, Germany, were used to form the development cohort. Details of the underlying patient population, as well as the assays and cutoffs used have been previously reported (5). Briefly, KTR received up to five doses of SARS-CoV-2 vaccine in case of sustained lack of sufficient serological response to vaccination at our institution, combined with either maintenance, reduction or pausing mycophenolic acid (MPA) for fourth and fifth vaccination. For the enzyme-linked immunosorbent assays (ELISA) for the detection of IgG antibodies against the S1 domain of the SARS-CoV-2 spike (S) protein in serum (Anti-SARS-CoV-2-ELISA (IgG), EUROIMMUN Medizinische Labordiagnostika AG, Lübeck, Germany), samples with a cutoff index ≥ 1.1 (in comparison to the previously obtained cut-off value of the calibrator) were considered positive, samples with a cutoff index ≥ 0.8, and < 1.1 were considered low positive, and samples with a cutoff index <0.8 were considered negative, as suggested by the manufacturer.

Alternatively, for the electrochemiluminescence immunoassay (ECLIA) (Elecsys, Anti-SARS-CoV-2, Roche Diagnostics GmbH, Mannheim, Germany) detecting human immunoglobulins, including IgG, IgA and IgM against the spike protein receptor binding domain (RBD), samples with ≥ 264 U/ml were considered to be positive as recommended by Caillard et al. (8, 9) Any non-zero antibody level below this cutoff was considered low positive, with limit of detection (LoD) being 0.4 U/mL.

For predictive modeling, we included data on third and fourth vaccination, since basic immunization has most likely been performed in most KTR patients already, and since only few patients received fifth vaccination so far.

**Figure 1:**
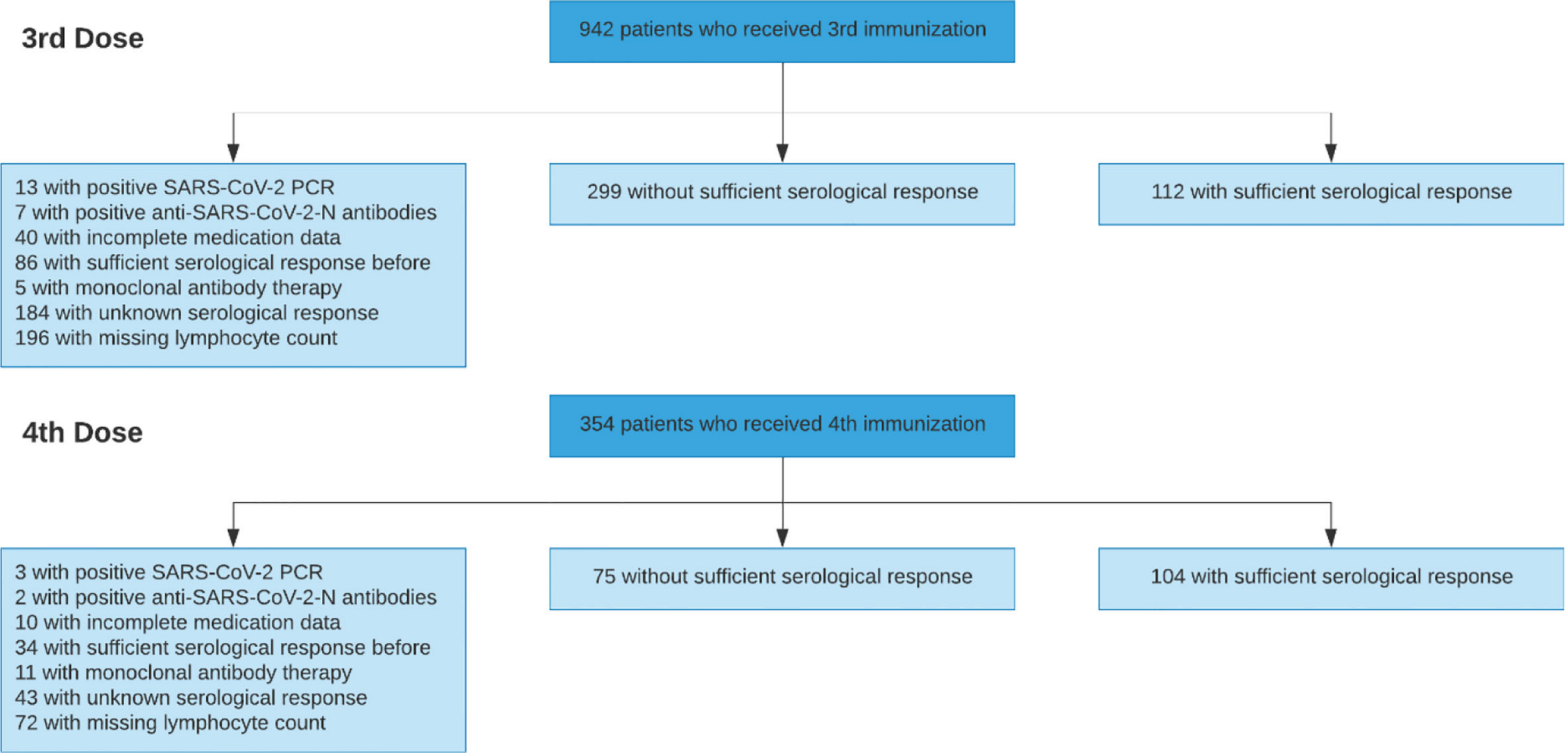
Patient flow diagram of the development cohort.

After applying all exclusion criteria summarized in **Table 1**, the development cohort comprised 590 vaccinations performed between December 2020 and January 2022 in 424 previously seronegative, COVID-naïve adult KTR (**Figure 1**). The Charité institutional review board approved this retrospective analysis (EA1/030/22).

**Table 1:** Inclusion and exclusion criteria regarding vaccinations.

Inclusion Criteria	
- Functioning kidney transplant at the time of vaccination
- Patient 18 years or older at the time of vaccination
- Third or fourth SARS-CoV-2 vaccination
- anti-SARS-CoV-2-S-protein antibodies below positivity cutoff before respective vaccination
- Follow-up anti-SARS-CoV-2-S-protein antibody measurement at least 14 days after vaccination
**Exclusion Criteria**
- SARS-CoV-2 vaccinations, which were performed before transplantation or after graft loss
- SARS-CoV-2 infection before the vaccination or before the measurement of the respective serological response as defined by - Positive SARS-CoV-2 RNA PCR - Positive anti-SARS-CoV-2-N-protein antibodies
- anti-SARS-CoV-2-S-protein antibodies above positivity cutoff before respective SARS-CoV-2 vaccination
- Monoclonal anti-SARS-CoV-2-S-protein antibody therapy before the measurement of the respective serological response
- Missing data on serological response before respective SARS-CoV-2 vaccination
- Missing data on serological response after respective SARS-CoV-2 vaccination
- Missing data on the assay used to measure serological response
- Missing data on immunosuppressive medication at the time of vaccination
- Missing lymphocyte count, eGFR, hemoglobin level

### Validation cohorts

We used four independent, international validation cohorts from outpatient transplant centers at University Hospital Düsseldorf, Germany (191 vaccinations in 137 KTR) (10, 11), Medical University Vienna, Austria (184 vaccinations in 184 KTR) (12), Strasbourg University Hospital, France (254 vaccinations in 229 KTR) (13, 14), Hotel Dieu Nantes, France (323 vaccinations in 269 KTR) (15). Detailed information about the validation cohorts are presented in **Items S1** **-** **Item S4** and patient selection including outcome frequencies are summarized separately for each validation cohort in **Figures S1**-**S4**. No sample size calculation was applicable for this *post-hoc* analysis.

### Outcome and predictors

The single outcome variable was a positive serological response defined by the maximum anti-SARS-CoV-2 spike (S) IgG or antibody level, measured at least 14 days after vaccination and before any further immunization event such as SARS-CoV-2 infection, passive or active immunization. Since different assays were used at different sites, details on the tests and the respective cutoffs used are provided for each validation cohort in **Item S1**-**S4**, which are summarized in **Table 2**. Generally, IgG or antibody positivity was determined based on the local laboratory’s positivity cutoff, mostly the one provided by the manufacturer. Especially for the ECLIA Elecsys assay different cutoffs were available and used. We chose to assess model performance for two cutoffs for this specific assay. First, we used the 0.8 U/mL cutoff provided by the manufacturer, yielding highest sensitivity in detecting patients with previous COVID-19. Second, a cutoff of 15 U/mL, which was initially suggested by the manufacturer to exhibit a positive predictive value of more than 99% for presence of neutralizing antibodies against the wild-type virus, was used (12). Contrary to the manufacturer’s designated use, our intention was to provide an alternative positivity cutoff, below which no neutralization against omicron variant occurs, but that is not as close to the LoD (0.4 U/mL) as the positivity cutoff provided by the manufacturer (0.8 U/mL). This alternative positivity cutoff definition was needed to test the hypothesis that the absence or low number of “low-positive” antibody levels before vaccination (below the positivity cutoff, but above the LoD) for this assay led to low performance in validation sets 2 and 4. While the cutoff of 15 U/mL is somewhat arbitrary, it meets both needs. First, it increases the percentage of low positive patients in validation set 4, and second, patients with antibody levels <50 U/mL in this assay show no neutralization against omicron BA.1, which most likely applies to omicron BA.2 as well (16, 17). Hence, adjusting the cutoff to 15 U/mL is compatible with the objective to identify patients without serological response to an additional vaccine dose corresponding best with a lack of neutralizing antibodies.

**Table 2:** Assays, as well as respective limit of detection and positivity cutoff used for each dataset.

Dataset + Assay	Assay (manufacturer)	Limit of Detection	Positivity Cutoff
Development	Anti-SARS-CoV-2 ELISA (IgG) assay (EUROIMMUN Medizinische Labordiagnostika AG, Lübeck, Germany)	0.8 index	≥1.1 index
Development	ECLIA Elecsys antibody assay (Roche Diagnostics GmbH, Mannheim, Germany)	0.4 U/mL	≥264 U/mL
Validation 1	Anti-SARS-CoV-2 QuantiVac ELISA (IgG) assay (EUROIMMUN Medizinische Labordiagnostika AG, Lübeck, Germany)	1 BAU/mL	≥35.2 BAU/mL
Validation 2	ECLIA Elecsys antibody assay (Roche Diagnostics GmbH, Mannheim, Germany)	0.4 U/mL	≥ 0.8 U/mL or ≥15 U/mL
Validation 3	CMIA SARS-CoV-2 IgG II Quant (Abbott, Rungis, France)	1 BAU/mL (7 AU/mL)	≥7 BAU/mL (50 AU/mL)
Validation 4	ECLIA Elecsys antibody assay (Roche Diagnostics GmbH, Mannheim, Germany)	0.4 U/mL	≥0.8 U/mL or ≥15 U/mL
Validation 4	LIAISON^®^ SARS-CoV-2 TrimericS IgG assay (Diasorin, Saluggia, Italy)	4.81 U/mL	≥33.8 BAU/mL
Validation 4	CMIA SARS-CoV-2 IgG II Quant (Abbott, Rungis, France)	7.8 AU/mL	≥50 AU/mL
Validation 4	NovaLisa SARS-CoV-2 IgG (Novatec Immundiagnostica GmbH, Dietzenbach, Germany)	1 U/mL	≥11 U/mL
Validation 4	Atellica^®^ IM SARS-CoV-2 IgG (sCOVG) (Siemens Healthineers, Erlangen, Germany)	0.5 index	≥2.0 index

Predictor variables in the data sets comprised 20 variables: four vaccination-specific, three demographic, one comorbidity, three transplantation-specific, five encoding medication, and four biomarkers (**Table S1**). From the initial 27 candidate predictor variables, seven were excluded during revision for the following reasons: treatment with azathioprine, mTOR inhibitor or rituximab in the last year were removed as predictor variable, since each variable was present in less than 5 subjects in the development dataset. Donor-specific anti-HLA antibodies were removed since they highly depend on mismatch status and medication adherence in the past. Anti-HB-S antibodies were removed since they depend on hepatitis B vaccination status, which was not available for most patients in the development cohort. Treatment with mycophenolic acid (MPA) was removed as predictor variable, since MPA dose contained the same information and was already used. White blood cell count was removed, since it is presumably less suitable to predict vaccination response than lymphocyte count, which was already a predictor variable (**Table S2** **)**.

### Missing data/imputation

For the development dataset, preliminary analysis showed that neither using data from patients without lymphocyte count, which was the most common missing laboratory value, nor imputation of missing laboratory values by multiple imputation (both of which yielded higher sample size) did add predictive accuracy for logistic regression and was therefore not followed for the main analysis (**Figure S5**; **Table S3**). After applying all exclusion criteria shown in **Table 1**, no missing values were present in the development dataset and no imputation methods were necessary.

For each validation set, we excluded vaccinations with missing data on serological response, missing information about the SARS-CoV-2 spike IgG or antibody assay used, missing immunosuppressive medication data, or missing estimated glomerular filtration rate (eGFR), lymphocyte count, or hemoglobin. We imputed the remaining variables to reduce the number of omitted cases due to missing values. Instead of multiple imputation, we used a more pragmatic approach and imputed either the most frequent value of the respective variable in the development dataset in case of binary or categorical variables, or the median (or mean) of the respective variable in the development dataset in case of numerical variables, as summarized in **Table 3**. This is the way a clinician would handle a missing value when using the online risk calculator, since those values are used as presets in the online calculator. In the validation cohorts, no data originating from a time after the respective vaccination was included to make predictions.

**Table 3:** Baseline characteristics of the development and validation cohorts.

	Development (Berlin)	Validation 1(Düsseldorf)	Validation 2 (Vienna)	Validation 3 (Strasbourg)	Validation 4 (Nantes) Cutoff 0.8U/mL	Validation 4 (Nantes) Cutoff 15U/mL
**Total vaccinations/patients**	590/424	191/137	184/184	254/229	254/211	323/269
**Vaccination**						
3rd/4th vaccinations	411/179	129/62	184/0	230/24	177/77	216/107
mRNA Vaccination	81.0% (478)	90.1% (173)	50.5% (93)	100% (254)	100% (254)	100% (323)
Median time since previous vaccination in days (IQR)	65 (51-92)	86 (79 - 140)	78 (57 - 90)	66 (49 - 65)	42 (31 - 93)	45 (31 - 92)
Baseline SARS-CoV-2 IgG low positive	6.8% (40)	40.1% (78)	0% (0)	40.2% (102)	14.6% (0)	33.1% (70)
**Demographics and Comorbidities**						
Female/male patients	38%/62%	32%/68%	41%/59%	41%/59%	47%/53%	46%/54%
Median age in years (IQR)	59 (47 - 69)	62 (54 - 68)	61 (54 - 70)	58 (50 - 68)	62 (52 - 69)	63 (52 - 70)
BMI in kg/m^2^	25.2 +/- 4.5	26.7 +/- 6.3	–	26.4 +/- 6.0	25.2 +/- 4.4	25.2 +/- 4.5
Diabetes	21.0% (124)	18.3% (35)	–	41.7% (106)	30.7% (78)	28.5% (92)
**Transplantation**						
Median transplant age in years (IQR)	7.8 (3.1 - 13.2)	4 (2.5 - 10)	4.4 (2.1 - 7.9)	5.2 (2.2 - 10.8)	4.1 (1.9 – 9.8)	4.6 (2.1 - 11.3)
Median time on dialysis in years (IQR)	3.0 (0.5 - 6.7)	3.1 (1 - 6)	–	2.2 (0.6 - 4.2)	1.3 (0 - 2.9)	1.3 (0 - 2.9)
Retransplantation	4.2% (25)	12.6% (24)	23.4% (43)	20.1% (51)	22.8% (58)	22.3% (72)
**Medication**						
CNI-based immunosuppression	87.3% (515)	95.8% (183)	91.3% (168)	93.7% (238)	85.8% (218)	85.7% (277)
Belatacept-based IS	11.2% (66)	4.2% (8)	7.6% (14)	3.2% (12)	9.5% (24)	8.7% (28)
MPA treatment	78.1% (461)	95.3% (182)	92.4% (171)	91.7% (233)	71.7% (182)	70.0% (226)
Median MPA-Dose in g MMF equivalent (IQR)	1.0 (0.5 - 1.5)	1.0 (1.0 - 1.5)	1.0 (1.0 - 2.0)	1.0 (1.0 - 1.0)	1.0 (0.0 - 1.0)	1.0 (0.0 - 1.0)
Steroid treatment	63.4% (374)	97.9% (187)	94.4% (174)	72.1% (183)	45.7% (115)	43.3% (140)
Treatment with more than 2 immunosuppressive drugs	45.4% (268)	95.3% (182)	91.3% (168)	69.7% (177)	27.0% (68)	25.7% (83)
**Laboratory values**						
Baseline eGFR mL/min/1.73m^2^	47.9 +/- 19.8	44.0 +/- 18.7	49.3 +/- 21.4	47.4 +/- 19.3	42.8 +/- 17.7	44.1 +/- 18.8
Lymphocyte count (/nL)	1.44 +/- 0.72	2.58 +/- 5.18	1.24 +/- 0.56	1.34 +/- 0.67	1.53 +/- 1.06	1.53 +/- 0.97
Hemoglobin (g/dL)	12.5 +/- 1.60	13.1 +/- 1.86	12.6 +/- 1.79	12.5 +/- 1.84	12.6 +/- 1.81	12.7 +/- 1.77
Median urine albumin-creatinine ratio in g/g (IQR)	0.030 (0.009 - 0.098)	0.034 (0.009 - 0.125)	0.035 (0.021 - 0.075)	0.046 (0.019 - 0.159)	0.031 (0.013 - 0.119)	0.030 (0.011 - 0.108)

All variables are reported as mean +/- standard deviation unless stated otherwise. IQR, interquartile range; mRNA, messenger ribonucleic acid; IgG, immunoglobulin G; BMI, body mass index; DSA, donor-specific anti human leukocyte antigen antibodies; CNI, calcineurin inhibitor; IS, immunosuppression; MPA, mycophenolic acid; MPA dose, mycophenolic acid dose; MMF, mycophenolate mofetil; mTORi, mammalian target of rapamycin inhibitor; eGFR, estimated glomerular filtration rate; anti-HBs, anti hepatitis B-surface-antigen immunoglobulin G antibodies.

Alternatively, we performed multiple imputations by chained equations employing the R package *mice* after pooling all validation datasets. Performing multiple imputation separately for each dataset was unfeasible, since for validation set 2, no data on BMI, time on dialysis and diabetes status were present. Pooling all validation sets and performing multiple imputation hereafter was one possibility to avoid this problem.

To compare median/mean imputation to other possibilities to deal with missing data, we additionally performed complete case analysis for validation sets 1, 3, and 4.

### Development and internal validation

Using the development cohort, we evaluated five models during internal validation. To perform model validation within the development cohort, a resampling approach was used by assigning 590 vaccinations randomly 100 times into training and test sets of 413 and 177 each (70:30 split). Each time, hyperparameter tuning, if applicable, and model fitting was performed on the respective training set, and performance metrics were assessed on the respective test set.

First, as baseline, we fit a logistic regression model with all candidate variables using the R function *glm*.

Second, we fit 2 logistic regression models with least absolute shrinkage and selection operator (LASSO) regularization using the packages *caret* and *glmnet* in R. The LASSO hyperparameter *λ*, which adjusts the tradeoff between model fit and model sparsity, was optimized for each training cohort with respect to the area under the receiver operating curve (AUC-ROC) using inner 5-fold cross-validation. We chose 2 different λ optimization criteria yielding 2 different models for each training cohort: (1) maximizing AUC-ROC (termed LASSO-Min model), and (2) penalty maximization while keeping the AUC-ROC within one standard error of the maximum AUC-ROC (termed LASSO-1SE model).

Third, we fit a random forest regression model using the package *randomForest* in R. We optimized the hyperparameter mtry by evaluating 15 random parameter combinations during two repeated 5-fold cross-validations within the training set. The value of mtry yielding the highest accuracy during cross-validation was used to fit the random forest on the respective training data.

Fourth, we fit a gradient boosted regression trees (GBRT) model using the *gbm* package. We used a tune grid with 4*8*3*1 hyperparameter combinations (n.trees: 300, 500, 700, 900; interaction.depth: 2, 4, 6, 8, 10, 12, 14, 16; shrinkage: 0.001, 0.01, 0.1; n.minobsinnode: 10) to optimize hyperparameters during two repeated 5-fold cross-validations within the training set. The combination yielding the highest normalized discounted cumulative gain during cross-validation was used to fit the GBRT on the respective training data.

We calculated median and mean performance during resampling for those five developed models. To evaluate the performance of the binary classification, we used Area Under the Curve of the Receiver Operator Characteristic (AUC-ROC), and confidence intervals (CI) in the resampling approach were determined from the empirical 2.5% and 97.5% quantiles of the performance on the 100 different test sets. Based on the threshold determined by the optimization criterion “closest.topleft” as provided in R package pROC (point with the least distance to [0,1] on the ROC-curve) during ROC-analysis, we calculated models’ sensitivity, specificity, accuracy, positive predictive value, and negative predictive value for each resampling step, again yielding median and empirical 95% CI.

### External validation and implementation

We chose LASSO-Min and LASSO-1SE for estimation of model coefficients in the entire development cohort, which were then used for external validation. The relationship between the hyperparameter *λ* that controls model sparsity and the AUC-ROC during inner 5-fold cross-validation is shown in **Figure S6**. We assessed the decision thresholds for classification by determining the “closest.topleft” threshold on the entire development cohort, each for the final 10-variable and the 20-variable model. These were used for classification during external validation, and are also provided in the online risk calculator after transforming them into risk probabilities according to the formula:


$$\text{P }\left({\text{x}}_{\text{t}}\right)\;=\;1\;/\;\left(\;1\;+\text{ exp }\left(\;-{\text{ x}}_{\text{t}}\right)\;\right)$$

where *x_t_* is the decision threshold.

For external validation, we calculated the aforementioned performance metrics on each validation cohort separately. Furthermore, 95% CIs in the external validation cohorts were determined by performing 1000-fold ordinary nonparametric percentile bootstrap, as the empirical 2.5%, and 97.5% quantiles of AUC, sensitivity, specificity, accuracy, positive predictive value, and negative predictive value based on the thresholds determined within the development cohort.

Additionally, we fitted LR, RF and GBRT on the development dataset and performed external validation after pooling all validation datasets. Decision thresholds for LR and GBRT were determined within the development cohort as described above, and decision threshold for RF was 0.5.

To make the prediction models publicly available, we created an online tool implementing the LASSO logistic regression models used for external validation, which can be assessed at https://www.tx-vaccine.com. For patients who meet one or more of the exclusion criteria, the risk calculator should not be used.

Statistical analysis was performed using R studio v.1.2.5042 and R version 4.1.2 (2021-11-01). The underlying code was made available at https://github.com/BilginOsmanodja/tx-vaccine. The datasets can be made available on request from the corresponding author.

This article was prepared according to the transparent reporting of a multivariable prediction model for individual prognosis or diagnosis (TRIPOD) statement and we provided a checklist in the supplement (18).

## Results

In total, 590 vaccinations (411 third vaccinations, and 179 fourth vaccinations) were used for development and internal validation, which is summarized together with outcome frequencies and reasons for exclusion in **Figure 1**.

Baseline characteristics of patients in the development and validation datasets including summary statistics of all variables are shown in **Table 3**.

### Model development and internal validation

Using the resampling approach outlined above, we fit five different models on each training set and evaluated their performance on the respective unseen test set during 100 resampling steps.

A logistic regression model employing all candidate variables served as a baseline. Using the two different λ optimization criteria outlined in “Methods”, the LASSO-Min and LASSO-1SE models were fitted. Additionally, two tree-based machine-learning approaches were studied - random forest (RF) and gradient boosted regression trees (GBRT).

**Figure 2:**
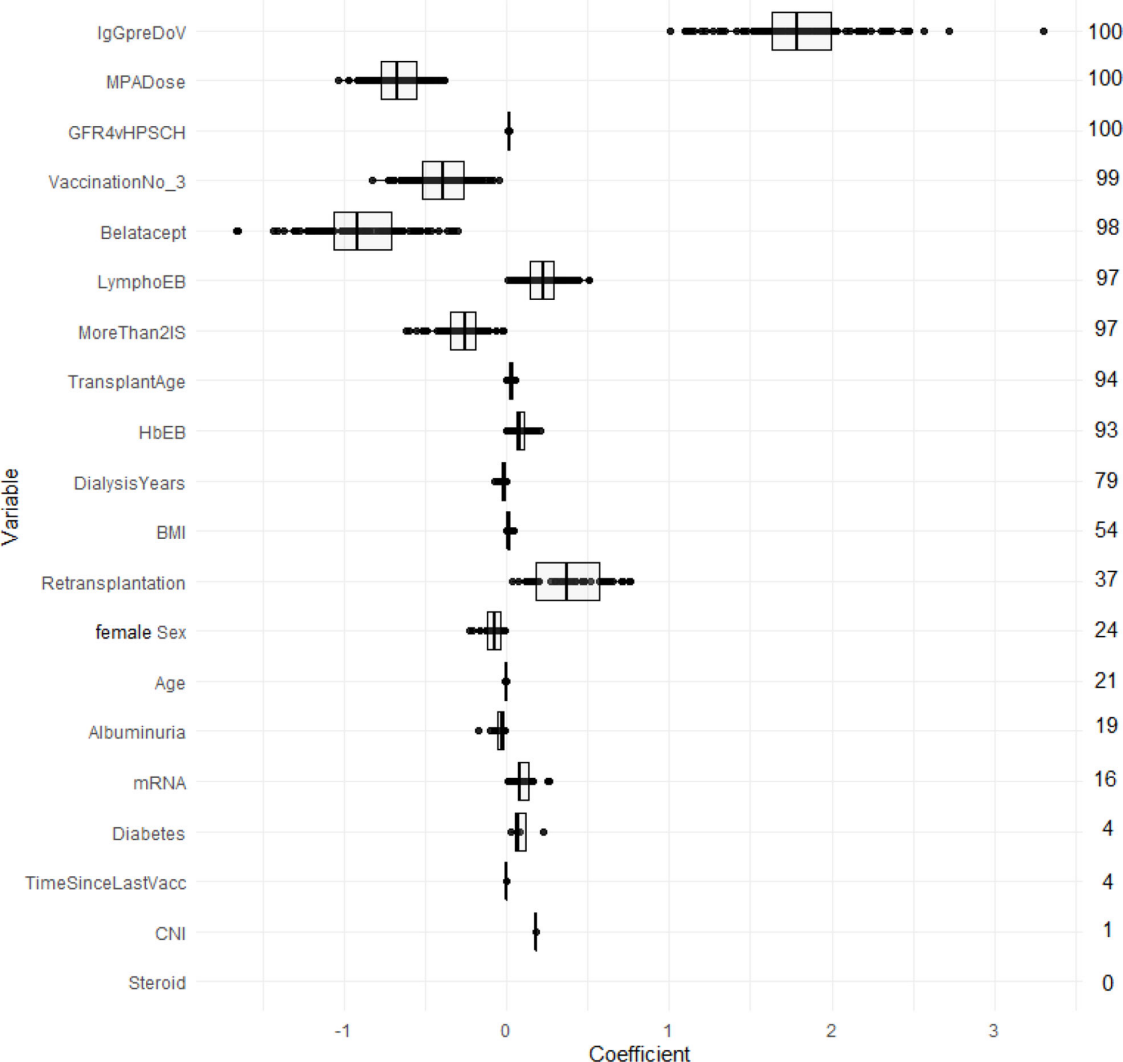
Estimated coefficients of the LASSO-1SE models summarized across 100 subsampling runs for unstandardized variables. Numbers on the right indicate the selection frequency (in percent) for the respective variable in 100 subsampling runs. Variables are ordered from top to bottom according to the selection frequency.

LASSO logistic regression selected in the majority of resampling runs 20, and 10 out of 20 potential predictors to yield the LASSO-Min and LASSO-1SE models, respectively. The regression coefficients, their variances, and the selection frequency of the predictors are shown in **Figure 2** and **Figure S7**.

**Figure 3:**
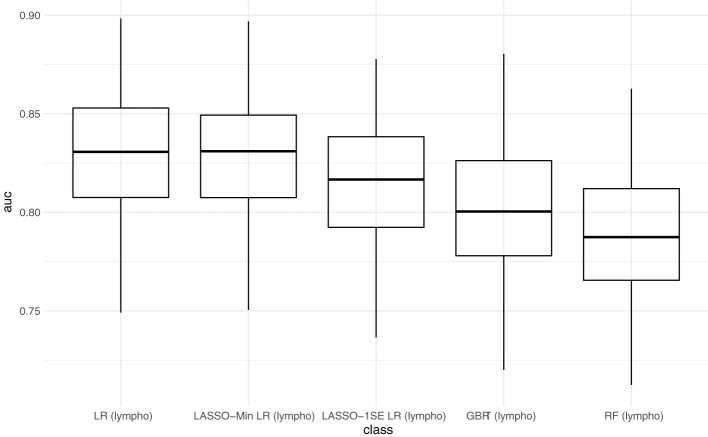
Predictive performance of the developed models (AUC) in internal validation. Each point represents the AUC-ROC during 1 out of 100 resampling steps. Horizontal lines within the box depict the median and the upper and lower horizontal lines depict upper and lower quartiles, respectively. LR - logistic regression, LASSO-Min LR - least absolute shrinkage and selection operator regularized logistic regression with lambda hyperparameter optimized to yield maximum AUC-ROC within an inner 5-fold cross validation in the training set. LASSO-1SE - least absolute shrinkage and selection operator regularized logistic regression with lambda hyperparameter increased from lambda-min, so that AUC-ROC stays within one standard error within an inner 5-fold cross validation in the training set. GBRT - gradient boosted regression trees. RF - random forest. lympho – including lymphocyte count as predictor variable.

**Figure 3** compares AUC-ROC of the 5 models on the unseen test sets during 100 resampling steps, and **Table 4** summarizes mean, and median performance metrics as well as 95% confidence intervals determined from empirical 2.5% and 97.5% quantiles during internal validation. Thresholds for binary classification were determined on the respective test set during each resampling step by performing ROC-analysis.

**Table 4:** Performance of five different models during internal validation.

Model Type	Mean/Median AUC (95%CI)	Mean/Median Sens (95%CI)	Mean/Median Spec (95%CI)	Mean/Median Acc (95%CI)	Mean/Median PPV (95%CI)	Mean/Median NPV (95%CI)
Logistic Regression	0.831/ 0.831 (0.786 - 0.879)	0.760/ 0.765 (0.651 - 0.839)	0.787/ 0.786 (0.700 - 0.870)	0.777/ 0.777 (0.715 - 0.819)	0.671/ 0.671 (0.566 - 0.785)	0.852/ 0.852 (0.787 - 0.900)
LASSO-Min	0.829/ 0.831 (0.784 - 0.879)	0.762/ 0.762 (0.672 - 0.852)	0.782/ 0.782 (0.693 - 0.880)	0.774/ 0.774 (0.712 - 0.825)	0.671/ 0.667 (0.562 - 0.778)	0.850/ 0.850 (0.792 - 0.902)
LASSO-1SE	0.814/ 0.817 (0.742 - 0.873)	0.734/ 0.736 (0.619 - 0.837)	0.779/ 0.780 (0.664 - 0.884)	0.762/ 0.763 (0.692 - 0.831)	0.661/ 0.659 (0.562 - 0.800)	0.836/ 0.839 (0.780 - 0.901)
Random Forest	0.789/ 0.787 (0.717 - 0.848)	0.503 0.503 (0.394 - 0.615)	0.898/ 0.897 (0.832 - 0.960)	0.753/ 0.757 (0.695 - 0.808)	0.744/ 0.737 (0.600 - 0.877)	0.758/ 0.764 (0.682 - 0.820)
GBM	0.802/ 0.800 (0.741 - 0.864)	0.730 0.729 (0.625 - 0.833)	0.767/ 0.768 (0.666 - 0.862)	0.753/ 0.751 (0.686 - 0.822)	0.646/ 0.646 (0.538 - 0.760)	0.832/ 0.831 (0.765 - 0.886)

AUC-ROC, as well as sensitivity (Sens), specificity (Spec), accuracy (Acc.), positive predictive value (PPV), negative predictive value (NPV) in the test set based on the best threshold during ROC-analysis. Mean, median and empirical 95% CI are derived from 100 resampling steps for each metric.

With respect to AUC-ROC, the LASSO-Min model - 0.831 (0.784 - 0.879) and the baseline logistic regression model - 0.831 (0.786 - 0.879) showed best performance during internal validation. Since the sparser LASSO-1SE model showed comparable predictive performance of 0.817 (0.742 - 0.873) with fewer variables, we chose to analyze both, LASSO-Min and LASSO-1SE regularized logistic regression models in depth during external validation.

### Model specification

Final risk equations were obtained by fitting LASSO-Min, and LASSO-1SE models on the complete development dataset, yielding a 20-variable and one 10-variable risk equation respectively. The intercept and regression coefficients of the final LASSO logistic regression models are shown in **Table 5**. Risk equations are provided in **Items S5** and **S6**, and are implemented as an online tool available at https://www.tx-vaccine.com.

**Table 5:** Final intercept and coefficients of the 20-variable (LASSO-Min), and 10-variable (LASSO-1SE) logistic regression model fitted on the entire development dataset, both of which are used for external validation.

	20-variable (LASSO-Min) model	10-variable (LASSO-1SE) model
Intercept	-2.907032206	-1.358548
Baseline SARS-CoV-2 IgG low positive (0/1)	3.413655483	1.772485
Third vaccination (0/1)	-0.671750504	-0.4788165
Female sex (0/1)	-0.307158368	–
Age (years)	-0.012892171	–
BMI in kg/m^2^	0.056292146	–
mRNA Vaccination (0/1)	0.296683923	–
Retransplantation (0/1)	1.320981616	–
Transplant age in years	0.074864392	0.02209966
Dialysis years	-0.074359667	-0.00005349
Diabetes (0/1)	0.227499203	–
Steroid (0/1)	-0.424257945	–
Belatacept (0/1)	-3.041854350	-0.5589842
CNI (0/1)	-0.938666068	–
MPA-Dose in g MMF equivalent	-1.421484726	-0.6303523
More than 2 immunosuppressants (0/1)	-0.184866365	-0.2549875
Days since previous vaccination	-0.003676502	–
Baseline eGFR mL/min/1.73m^2^	0.025117386	0.009467306
Lymphocyte count (/nL)	0.469212486	0.2598442
Hemoglobin (g/dL)	0.206815906	0.0554962
Albuminuria (g/g creatinine)	-0.269263716	–

### External validation

After applying all exclusion criteria and performing imputation of missing variables, we evaluated both risk equations in the four independent validation datasets. Since predictive performance during external validation was comparable for both models, in the following we report on the sparser 10-variable model. Results of external validation of the 20-variable model are summarized in **Table S4** and **Figure S7**.

**Figure 4:**
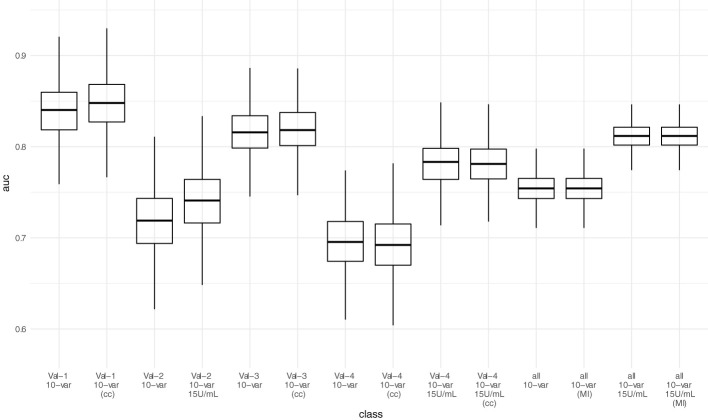
Predictive performance (AUC-ROC) of the 10-variable model in external validation. Each point represents the AUC-ROC in 1 out of 1000 bootstrap samples. Horizontal lines within the box depict the median and the upper and lower horizontal lines depict upper and lower quartiles, respectively. To assess the impact of the mean/median imputation method chosen, we also provide model performance when performing complete case analysis (cc) for validation sets 1, 3, and 4. For validation set 2, due to missing variable “Dialysis years” for all patients, no complete case analysis could be performed. Additionally, we performed multiple imputation (MI) in the pooled validation datasets (all) and assessed model performance here as well. Val – Validation cohort, 10-var – 10-variable model, all – all validation sets pooled, cc – complete case analysis, MI – multiple imputation.

AUC-ROC of the sparser 10-variable model during external validation was 0.840 (0.777 - 0.897) for validation set 1, 0.719 (0.641 - 0.790) for validation set 2, 0.816 (0.763 - 0.862) for validation set 3, and 0.696 (0.629 - 0.758) for validation set 4, yielding an AUC-ROC of 0.754 (0.722 - 0.784) when merging all validation sets **(** **Figure 4** **)**. Sensitivity, specificity, accuracy, positive predictive value, and negative predictive value using the thresholds determined during ROC-analysis in the development dataset are summarized in **Table 6**. The decision thresholds used for external validation are also provided in the online risk calculator to guide physicians’ decision as well in **Items S5** and **S6**.

**Table 6:** Performance of the 10-variable model during external validation.

Model Type	AUC point estimate (95% CI)	Sens point estimate (95% CI)	Spec point estimate (95% CI)	Acc point estimate (95% CI)	PPV point estimate (95% CI)	NPV point estimate (95% CI)
Validation 1 10-variable	0.840 (0.777 - 0.897)	0.769 (0.667 - 0.868)	0.675 (0.589 - 0.758)	0.707 (0.639 - 0.775)	0.551 (0.448 - 0.653)	0.852 (0.774 - 0.913)
Validation 1 10-variable (complete case)	0.848 (0.781 - 0.905)	0.754 (0.636 - 0.865)	0.696 (0.614 - 0.778)	0.715 (0.642 - 0.782)	0.535 (0.423 - 0.657)	0.857 (0.785 - 0.922)
Validation 2 10-variable (cutoff 0.8 U/mL)	0.719 (0.641 - 0.790)	0.127 (0.051- 0.214)	0.933 (0.881 - 0.972)	0.630 (0.560 - 0.696)	0.533 (0.286 - 0.750)	0.640 (0.565 - 0.707)
Validation 2 10-variable (cutoff 15 U/mL)	0.741 (0.663 - 0.808)	0.128 (0.029 - 0.243)	0.917 (0.869 - 0.959)	0.750 (0.685 - 0.804)	0.286 (0.091 - 0.522)	0.798 (0.736 - 0.853)
Validation 3 10-variable	0.816 (0.763 - 0.862)	0.715 (0.639 - 0.791)	0.738 (0.655 - 0.814)	0.727 (0.672 - 0.783)	0.721 (0.638 - 0.802)	0.733 (0.652 - 0.805)
Validation 3 10-variable (complete case)	0.818 (0.763 - 0.870)	0.707 (0.624 - 0.781)	0.736 (0.662 - 0.815)	0.720 (0.665 - 0.776)	0.719 (0.645 - 0.794)	0.725 (0.648 - 0.797)
Validation 4 10-variable (cutoff 0.8 U/mL)	0.696 (0.629 - 0.758)	0.634 (0.556 - 0.707)	0.626 (0.538 - 0.716)	0.630 (0.575 - 0.693)	0.710 (0.630 - 0.780)	0.544 (0.462 - 0.632)
Validation 4 10-variable (cutoff 0.8 U/mL – cc)	0.692 (0.622 - 0.758)	0.633 (0.559 - 0.709)	0.625 (0.525 - 0.717)	0.630 (0.571 - 0.689)	0.708 (0.633 - 0.784)	0.539 (0.448 - 0.631)
Validation 4 10-variable (cutoff 15 U/mL)	0.783 (0.730 - 0.828)	0.775 (0.718 - 0.828)	0.603 (0.513 - 0.680)	0.709 (0.656 - 0.759)	0.761 (0.703 - 0.814)	0.622 (0.534 - 0.708)
Validation 4 10-variable (cutoff 15 U/mL – cc)	0.781 (0.725 - 0.825)	0.775 (0.714 - 0.833)	0.602 (0.520 - 0.695)	0.708 (0.658 - 0.755)	0.760 (0.701 - 0.815)	0.623 (0.531 - 0.701)
**Overall performance** **10-variable**	**0.754 (0.722 - 0.784)**	**0.593 (0.545 - 0.641)**	**0.743 (0.705 - 0.777)**	**0.673 (0.641 - 0.706)**	**0.666 (0.617 - 0.711)**	**0.679 (0.637 - 0.720)**
Overall performance 10-variable (MI)	0.754 (0.722 - 0.784)	0.593 (0.545 - 0.641)	0.743 (0.705 - 0.777)	0.673 (0.641 - 0.706)	0.666 (0.617 - 0.711)	**0.679 (0.637 - 0.720)**
**Overall performance** **10-variable** **(cutoff 15 U/mL)**	**0.812 (0.784 - 0.836)**	**0.698 (0.654 - 0.737)**	**0.741 (0.702 - 0.775)**	**0.722 (0.691 - 0.749)**	**0.687 (0.642 - 0.727)**	**0.750 (0.713 - 0.784)**
Overall performance 10-variable (cutoff 15 U/mL – MI)	0.812 (0.784 - 0.836)	0.698 (0.654 - 0.737)	0.741 (0.702 - 0.775)	0.722 (0.691 - 0.749)	0.687 (0.642 - 0.727)	0.750 (0.713 - 0.784)

AUC-ROC, as well as sensitivity (Sens), specificity (Spec), accuracy (Acc.), positive predictive value (PPV), negative predictive value (NPV) assessed on each validation set. To assess the impact of the mean/median imputation method chosen, we also provide model performance during complete case analysis for validation sets 1, 3, and 4. For validation set 2, due to missing variable “Dialysis years” for all patients, no complete case analysis could be performed. Additionally, we performed multiple imputation in the pooled validation datasets and assessed model performance here as well. The threshold was derived during ROC-analysis on the development dataset. To provide 95% CI, empirical 2.5% and 97.5% quantiles of the respective metric are provided after performing a 1000-fold nonparametric ordinary bootstrapping with each validation set. Overall performance in the pooled validation sets are bold-faced. cc - complete case analysis. MI – multiple imputation.

### Implementation and cutoff definition

Performance in the validations sets 2 and 4 was poorer than in the development as well as in the other two validation sets. We suspected the positivity cutoff of 0.8 U/mL provided by the manufacturer for the ECLIA Elecsys assay as one main reason. Since it is close to the LoD (0.4 U/mL), no or small fraction of “low positive” antibody levels (values above the LoD and below positivity cutoff) before vaccination are present in both validations sets (**Table 3**), which is different to both other validation sets and the development dataset. Since a low positive antibody level before vaccination is an important predictor of serological response (**Table 5**), we adjusted the positivity cutoff to 15 U/mL arbitrarily for two reasons. First, to test the hypothesis that cutoff definition is a reason for lower performance. Second, to provide data that an implementation of this model is feasible independent of the assay used. Our proposed implementation strategy for the prediction model is to identify patients, who will not respond to an additional vaccine dose, and to offer those patients passive immunization. Hence, using any other cutoff below which no neutralization against omicron occurs, is compatible with this strategy under the circumstances of omicron-dominance. We arbitrarily use an alternative positivity cutoff of 15 U/mL for this respective assay, since it has already been proposed by the manufacturer before.

When adjusting the cutoff to 15 U/mL for the ECLIA Elecsys assay, AUC-ROC increased to 0.741 (0.663 - 0.808) for validation set 2, and 0.783 (0.730 - 0.828) for validation set 4, yielding an overall AUC-ROC of 0.812 (0.784 - 0.836) after merging all validation sets. With the decision threshold assessed in the development dataset, the negative predictive value is 0.75 (0.713 - 0.784).

Neither complete case analysis nor multiple imputation in the pooled validation cohort led to relevant differences in predictive performance for the 20-variable and 10-variable LASSO LR models **(** **Table 6**; **Table S4** **)**.

### Tree-based models

**Figure 5:**
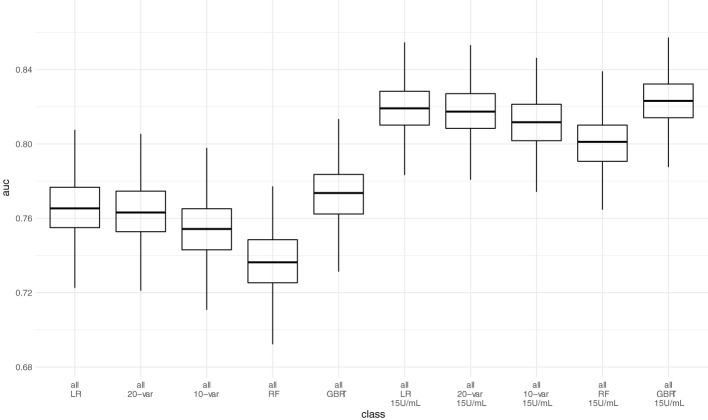
Predictive performance of the 10-variable, 20-variable LASSO logistic regression, logistic regression (LR), random forest (RF) and gradient boosted regression tree (GBRT) models on the pooled validation set comprised of all 4 validation sets with cutoff 0.8 U/mL and 15 U/mL for the Elecsys assay. 10-var – 10-variable LASSO LR model, 20-var – 20-variable LASSO LR model, all – all validation sets pooled.

Next, we assessed model performance of all 5 models in the pooled validation set, using both the cutoff of 0.8 U/mL and 15 U/mL for the Elecsys assay, respectively **(** **Figure 5** **)**. For the 15 U/mL cutoff, GBRT showed AUC-ROC of 0.823 (0.795 - 0.849), which was slightly better than LR 0.819 (0.791 - 0.847) and the LASSO-Min model 0.817 (0.790 - 0.845). The LASSO-1SE model with 0.812 (0.784 - 0.836) was still better than the RF model - 0.801 (0.771 - 0.828). All results are summarized in **Table 7**. Analysis of feature importance for the tree-based models revealed that for the RF model, low positive antibody titer, MPA dose, belatacept treatment, vaccination number, and transplant age were the five most important variables, while diabetes, time since vaccination and sex were the least important variables. For the GBRT model, MPA dose, low positive antibody titer, eGFR, lymphocyte count and transplant age were the most important variables, while diabetes, CNI treatment, and mRNA-based vaccine were the least important variables. When comparing the 10 most important variables from RF or GBRT to the LASSO-1SE model, RF as well as GBRT had 8 out of 10 variables in common with the 10-variable LASSO-1SE model **(** **Table 8** **).**

**Table 7:** Performance of all five different models during external validation on the pooled validation datasets.

Model Type	AUC	Sens	Spec	Acc	PPV	NPV
Logistic Regression 0.8 U/mL	0.765 (0.734 - 0.797)	0.711 (0.668 - 0.753)	0.648 (0.605 - 0.690)	0.677 0.646 - 0.708)	0.634 0.594 - 0.677)	0.721 (0.680 - 0.762)
20-variable LASSO LR 0.8 U/mL	0.763 (0.731 - 0.794)	0.691 (0.646 - 0.735)	0.666 (0.623 - 0.709)	0.677 0.647 - 0.710)	0.641 0.597 - 0.684)	0.714 (0.672 - 0.756)
10-variable LASSO LR 0.8 U/mL	0.754 (0.722 - 0.784)	0.593 (0.545 - 0.641)	0.743 (0.705 - 0.777)	0.673 0.641 - 0.706)	0.666 0.617 - 0.711)	0.679 (0.637 - 0.720)
Random Forest 0.8 U/mL	0.736 (0.704 - 0.769)	0.537 (0.489 - 0.585)	0.812 (0.778 - 0.846)	0.684 (0.653 - 0.716)	0.712 (0.662 - 0.758)	0.670 (0.662 - 0.758)
GBRT 0.8 U/mL	0.774 (0.741 - 0.802)	0.650 (0.603 - 0.695)	0.737 (0.697 - 0.775)	0.695 (0.666 - 0.727)	0.681 (0.633 - 0.726)	0.710 (0.669 - 0.749)
Logistic Regression 15 U/mL	0.819 (0.791 - 0.847)	0.790 (0.751 - 0.829)	0.638 (0.600 - 0.679)	0.707 (0.676 - 0.736)	0.642 (0.596 - 0.680)	0.788 (0.750 - 0.827)
20-variable LASSO LR 15 U/mL	0.817 (0.790 - 0.845)	0.780 (0.740 - 0.821)	0.664 (0.624 - 0.704)	0.716 (0.687 - 0.746)	0.655 (0.610 - 0.695)	0.787 (0.748 - 0.826)
10-variable LASSO LR 15 U/mL	0.812 (0.784 - 0.836)	0.698 (0.654 - 0.737)	0.741 (0.702 - 0.775)	0.722 (0.691 - 0.749)	0.687 (0.642 - 0.727)	0.750 (0.713 - 0.784)
Random Forest 15 U/mL	0.801 (0.771 - 0.828)	0.651 (0.605 - 0.695)	0.809 (0.776 - 0.840)	0.737 (0.709 - 0.765)	0.735 (0.692 - 0.777)	0.739 (0.703 - 0.773)
GBRT 15 U/mL	0.823 (0.795 - 0.849)	0.745 (0.705 - 0.788)	0.731 (0.695 - 0.767)	0.737 (0.708 - 0.765)	0.693 (0.649 - 0.732)	0.779 (0.742 - 0.815)

AUC-ROC, as well as sensitivity (Sens), specificity (Spec), accuracy (Acc.), positive predictive value (PPV), negative predictive value (NPV) assessed on the pooled validation set, once employing the cutoff of 0.8 U/mL and once employing the cutoff of 15 U/mL for the Elecsys assay. The decision threshold was derived during ROC-analysis on the development dataset. To provide 95% CI, empirical 2.5% and 97.5% quantiles of the respective metric are provided after performing a 1000-fold nonparametric ordinary bootstrapping with each validation set.

**Table 8:** Comparison of feature importance of random forest (RF), gradient boosted regression trees (GBRT), and variable selection in the LASSO-1SE model.

	Random Forest – Mean Decrease Accuracy	GBRT – Relative Influence	LASSO-1SE model (10-variables)
Baseline SARS-CoV-2 IgG low positive (0/1)	39.216473	10.94581396	1.772485
MPA-Dose in g MMF equivalent	30.188770	16.56390709	-0.6303523
Transplant age in years	10.848819	9.45975758	0.02209966
Third vaccination (0/1)	21.775882	5.43038673	-0.4788165
Baseline eGFR mL/min/1.73m^2^	9.018273	10.35407127	0.009467306
Lymphocyte count (/nL)	6.344629	9.46973950	0.2598442
Belatacept (0/1)	23.579373	5.16012305	-0.5589842
More than 2 immunosuppressants (0/1)	10.293539	1.76980409	-0.2549875
Hemoglobin (g/dL)	4.199420	7.10326964	0.0554962
Dialysis years	4.356144	5.42538246	-0.00005349
CNI (0/1)	8.862467	0.04204069	–
mRNA Vaccination (0/1)	5.530626	0.13629129	–
Days since previous vaccination	-2.417074	5.73285303	–
BMI in kg/m^2^	1.249847	5.42081191	–
Albuminuria (g/g creatinine)	3.941902	2.16932962	–
Retransplantation (0/1)	1.992805	0.69025148	–
Steroid (0/1)	4.321701	0.18964166	–
Age (years)	1.641021	3.50130734	–
Female sex (0/1)	-2.429416	0.40048841	–
Diabetes (0/1)	-1.571995	0.03472919	–

The feature importance of the random forest (RF) model was assessed by calculating mean decrease in accuracy. For GBRT, relative influence is shown. Variables for RF and GBRT are highlighted according to their importance in green (1-5), light green (6-10), yellow (11-15), and red (16-20).

To assess how variable selection based on feature importance influences model performance, we selected the 10 most important variables for both RF and GBRT, retrained the models and performed external validation in the pooled validation dataset. Both, the 10-variable RF and the 10-variable GBRT yielded the same AUC-ROC during external validation as the respective 20-variable models - 0.823 (0.795 - 0.849) for GBRT, and 0.801 (0.771 - 0.828) for RF.

## Discussion

In this article, we present the development, internal and external validation of a 10-variable LASSO regularized logistic regression model for prediction of serological response to the third and fourth dose of SARS-CoV-2 vaccine in previously seronegative, COVID-19-naïve KTR.

It shows good discrimination of KTR exhibiting serological response both in a rigorous resampling approach in the development cohort and in four independent validation cohorts with an overall AUC-ROC of 0.812, and a negative predictive value of 0.75 based on a decision threshold established within the development dataset. Available online as a risk calculator at https://www.tx-vaccine.com and embedded into the proposed implementation strategy, it can assist physicians in choosing between different immunization strategies, namely, additional vaccination with or without adaption of immunosuppressive therapy, or pre-exposure prophylaxis with monoclonal anti-SARS-CoV-2-(S) antibodies.

While this is the first, online available risk calculator to predict seroconversion in response to third and fourth vaccination, there are already models predicting seroconversion after two vaccine doses.

Frölke et al. describe a sparse 6-variable model, where increased age, lower lymphocyte count, lower estimated glomerular filtration rate (eGFR), shorter time after transplantation, not using steroids and the use of mycophenolate mofetil/mycophenolic acid (MMF/MPA) are predictors of non-seroconversion (with a cutoff of 10 BAU/mL). This is completely in line with our own findings. The performance in the development (n=215) and rather small validation cohort (n=73) are promising (AUC-ROC 0.83 and 0.84, respectively). In a larger, second validation cohort, for which an adapted model without lymphocyte count was used, the performance drops to AUC-ROC 0.75, which emphasizes that lymphocyte count is an important predictor (19). Still, this model is easy to use, and shows apparently more stable performance than the other model available, which is provided by Alejo et al. They use a gradient boosting algorithm, and identified mycophenolate mofetil (MMF) use, shorter time since transplant, and older age as strongest predictors of non-seroconversion, which is in line with our findings as well. Since the model shows good predictive performance on the development dataset (AUC-ROC 0.79), but poor performance in an external validation cohort (AUC-ROC 0.67), it can be suspected that the model is overfitted (20). This is further supported when using the online tool the authors provide at http://www.transplantmodels.com/covidvaccine/, where small changes, e.g. in patient age, show great changes in vaccine response probability. Another reason for worse performance could be that not only kidney transplant recipients are included. Therefore, not only is the cohort more heterogeneous, but important predictors such as eGFR are missing.

Our own data show that GBRT can achieve comparable performance in internal and external validation, when overfitting is limited by hyperparameter tuning within the development cohort. Nevertheless, since the GBRT model did not substantially outperform LASSO logistic regression models, but is more complex and less transparent, we chose not to implement the GBRT model in the online calculator. These findings are in line with the statistical literature showing no benefit of machine learning methods over logistic regression for clinical prediction models (21).

From a biomedical point of view, serological response is only one half of immune response to vaccination and is complemented by T-cell response. However, neutralizing anti-SARS-CoV-2-(S) antibodies are pathophysiologically and epidemiologically established to offer protection from severe disease (9, 22), which is also supported by the protection offered by monoclonal antibodies against SARS-CoV-2 applied for prophylaxis and treatment (23, 24).

Yet, after the emergence of the omicron variants, neutralization antibody levels against omicron variant show 25.7-fold to 58.1-fold reduction in sera of healthy vaccinated subjects in comparison to wild-type (25). Consequently, antibody levels that ensure neutralization, increased from >264 U/mL for alpha variant (8, 9) to >2000 U/mL for omicron (16), making the interpretation of antibody levels more difficult than before.

Despite these uncertainties, it seems intolerable to leave patients without any humoral protection whatsoever. Hence, in patients without serological response to basic immunization, physicians and patients need to decide between additional active vaccination with or without adapting immunosuppressive medication, and pre-exposure prophylaxis with monoclonal antibodies (26).

Since negative predictive value was above 0.75, when merging all validation sets, we suggest the following implementation strategy: for patients, who are likely not to respond to additional SARS-CoV-2 vaccination according to the prediction model, pre-exposure prophylaxis with monoclonal antibodies exhibiting neutralizing capacity against omicron BA.4/5 should be administered to ensure timely protection (27–30). In patients, who are likely to respond according to the prediction model, there is still a chance that these patients will not reach antibody levels, which ensure neutralizing capacity against omicron variants. For these patients, both, repeated vaccination and monoclonal antibody prophylaxis are feasible and should in our view be chosen depending on the risk for severe disease course.

### Strengths and limitations

We provide a rigorously developed and validated prediction model, which is provided as an online risk calculator to support kidney transplant physicians when deciding upon the immunization strategy for their patients. Especially, the estimated effects of adaptions in immunosuppressive medication can be evaluated, e.g. reducing or pausing MPA dose, or switching from belatacept to calcineurin inhibitor.

While belatacept treatment and MPA dose have been reported as negative predictors of serological vaccine response throughout the literature (10), it is still unclear, whether modulation of immunosuppression, and especially pausing MPA can increase serological response to SARS-CoV-2 vaccination, since most data originate from observational or small non-randomized controlled trials (5, 31–33).

Since the data from the development cohort indicate that pausing MPA increases serological response to fourth vaccination, this hypothetical benefit is introduced into the model. Hence, if used to guide modulation of immunosuppression and specifically pausing MPA, the calculator must be used with caution, since the hypothetical effect on serological response comes at a potential risk of anti-HLA antibody formation and rejection.

Additionally, predictions can be suspected to be less accurate in cohorts, where no modulation of immunosuppression are performed around fourth vaccination.

Regarding the general sparsity of data on vaccine response to third and fourth dose in KTR, we analyze extensive datasets for development and validation, and hereby provide representative evaluation of real-life model performance. While performance of the 20-variable model was slightly better, it is also more impractical due to more variables, which is why we chose to report mainly on the sparser 10-variable model. The same applies for the 20-variable GBRT model, which was not implemented in the online calculator. When compared to other models reported for this purpose, it is the first to predict serological response to third and fourth vaccination, and shows the most promising performance during external validation.

Several limitations have to be considered: first, this model only predicts serological response and does not include information about T-cell response. However, we have shown before that more than 85% of KTR have SARS-CoV-2 specific CD4+ T-cell response after three vaccinations, which was not increased by fourth vaccination, while serological response rates increase with additional vaccinations (4, 5). This is the rationale, why especially serological response rate can and should be increased to improve protection from SARS-CoV-2 infection in KTR.

Since evidence of antibody level cutoffs that ensure neutralization of or protection from omicron is sparse, we chose not to make any predictions for this endpoint. Instead, we provide an implementation strategy that makes best use of the model’s prediction without making far-reaching assumptions about protective antibody levels against omicron.

Still, one major limitation becomes evident for validation sets 2 and 4, where predictive performance was only moderate when using the positivity cutoff of 0.8 U/mL for the ECLIA Elecsys assay. As outlined above, increasing the positivity cutoff to 15 U/mL is compatible with the proposed implementation, and leads to improved performance for two reasons.

First, since the cutoffs in the development dataset were based on protective levels against alpha variant for the ECLIA Elecsys assay (264 U/mL), predictive performance is expectably poorer when predicting positivity with a 0.8 U/mL cutoff. Second, with a cutoff of 0.8 U/mL for the ECLIA Elecsys assay, only few patients have low positive antibody levels before vaccination (above LoD, but below the positivity cutoff). Since this is an important predictor in both models and provides important information about the actual immunological status, loss of performance can be expected when this information is missing. This is further supported by the fact that after adapting the cutoff from 0.8 U/mL to 15 U/mL, in validation set 2, where all pre-vaccination antibody levels were below 0.4 U/mL, the performance only increased slightly (AUC-ROC 0.719 to 0.741), but in validation set 4, where the percentage of low positive patients increased from 14.6% (most of which were due to the other assays used in this dataset) to 33.1%, the performance increased markedly (AUC-ROC 0.696 to 0.783).

On the contrary, since the percentage of low positive antibody levels in the development dataset is 6.8%, performance in cohorts, where this rate is close to 100%, could potentially worsen as well.

Other possible reasons for different performance are the study design of validation set 2, which was a randomized clinical trial, with outcome assessment between days 29 and 42, whereas in the development and other validation sets, the maximum antibody level after the respective vaccination was chosen, independent of the time passed. Additionally, validation set 2 was the one with the highest proportion of adenoviral vaccine, which however, did not show any difference in serological response in the respective trial (12).

Worth discussing is the mean/median imputation method used, which ensures that performance assessed during external validation is comparable to real-life performance of the risk calculator provided. We show that neither performing complete case analysis nor multiple imputation in the validation cohorts substantially changes predictive performance.

Other limitations arise from the different immunization strategies used, which lead to different seroconversion rates and have influence on model performance as well.

Last, some immunosuppressive regimens have low frequency below 1% in the development cohort (such as rituximab, mTOR inhibitor, and azathioprine treatment), which limits applicability for these patients.

## Conclusion

We provide the first, online available calculator to predict vaccine response to third or fourth vaccination in previously seronegative, COVID-19-naïve KTR. It can guide decisions whether to modulate immunosuppressive therapy before additional active vaccination, or to perform passive immunization to improve protection against COVID-19.

## Data availability statement

The raw data supporting the conclusions of this article will be made available by the authors, without undue reservation.

## Ethics statement

The studies involving human participants were reviewed and approved by Ethics Comittee of Charité Universitätsmedizin Berlin. Written informed consent for participation was not required for this study in accordance with the national legislation and the institutional requirements.

## Author contributions

BO and SR conceived of the presented idea. BO performed data analysis and implemented the risk calculator. SR performed data visualization and project coordination. FG and MM assisted in data preparation of the development dataset. JS, MK, LCR, AH, RR-S, RO, IB, SC, CM, CK, and GB provided validation datasets. BO and SR wrote the manuscript. KB, ES, JS, MK, AH, RR-S, IB, SC, and CM provided significant intellectual input during the conception and development of the article. All authors commented and reviewed the final manuscript.

## Acknowledgments

Eva Schrezenmeier is a participant in the BIH-Charité Clinician Scientist Program funded by the Charité - Universitätsmedizin Berlin and the Berlin Institute of Health. We thank Jürgen Dönitz, Johannes Raffler, Ulla Schultheiss, and Helena U. Zacharias on behalf of the CKDapp team for providing their template for an online risk calculator.

## Conflict of interest

The authors declare that the research was conducted in the absence of any commercial or financial relationships that could be construed as a potential conflict of interest.

## Publisher’s note

All claims expressed in this article are solely those of the authors and do not necessarily represent those of their affiliated organizations, or those of the publisher, the editors and the reviewers. Any product that may be evaluated in this article, or claim that may be made by its manufacturer, is not guaranteed or endorsed by the publisher.

## Abbreviations

AUC-ROC, area under the curve of the receiver operator characteristic; CI, confidence intervals; CNI, calcineurin inhibitor; COVID-19, coronavirus disease 2019; GBRT, gradient boosted regression trees; ECLIA, electrochemiluminescence immunoassay; eGFR, estimated glomerular filtration rate; ELISA, enzyme-linked immunosorbent assays; KTR, kidney transplant recipients; LASSO, least absolute shrinkage and selection operator; LoD, limit of detection; LR, logistic regression; MPA, mycophenolic acid; N, nucleocapsid protein; RBD, receptor binding domain; S, spike protein; SARS-CoV-2, severe acute respiratory syndrome coronavirus 2; SOT, solid organ transplantation; TCMR, T cell mediated rejection; TRIPOD, transparent reporting of a multivariable prediction model for individual prognosis or diagnosis.
